# Plasma Interleukin-6 Level: A Potential Prognostic Indicator of Emergent HBV-Associated ACLF

**DOI:** 10.1155/2021/5545181

**Published:** 2021-11-11

**Authors:** Zhe-Bin Wu, Yu-Bao Zheng, Ke Wang, Zhi-Shuo Mo, Xu Zhen, Ying Yan, Zhi-Liang Gao

**Affiliations:** Deparment of Infectious Diseases, The Third Affiliated Hospital of Sun Yat-Sen University, Guangzhou, China

## Abstract

**Objective:**

To identify markers that predict the progression to hepatitis B virus-associated acute-on-chronic liver failure (HBV-ACLF).

**Methods:**

We recruited 125 patients with chronic hepatitis B (CHB) between September 2013 and March 2017. During hospitalization, 25 patients progressed to LF and were classified as the LF group, while the remaining 100 patients were classified as the non-LF (NLF) group. We compared the kinetic changes in clinical and immune indicators including age, total bilirubin level, prothrombin time, model for end-stage liver disease score, interleukin (IL)-6, IL-8, and IL-10 cytokine levels, and number of T helper 17 and regulatory T cells between groups to determine their association with progression to HBV-ACLF. The prognostic value of clinical and immune indicators was determined using the area under the receiver operating characteristic curve (AUC) value.

**Results:**

Cox regression analysis suggested that the plasma IL-6 level could predict CHB progression to HBV-ACLF (relative risk = 1.082, 95% confidence interval: 1.006–1.164; *P*=0.034). The AUC value, sensitivity, and specificity of baseline IL-6 level for predicting HBV-ACLF were 82.63%, 83.3%, and 82.9%, respectively (*P*=0.001).

**Conclusion:**

A high plasma IL-6 level in CHB patients could be an early biomarker for HBV-ACLF.

## 1. Introduction

Chronic hepatitis B virus (HBV) infection may potentially precipitate to fatal liver diseases, thereby imposing a heavy global public health burden. As high as 80% of liver failure (LF) cases are related to chronic HBV infection in China [1]. Asymptomatic chronic HBV can be rapidly exacerbated and cause liver injury and frequently progress to acute-on-chronic LF (ACLF) [2]. Despite the high mortality rate of HBV-ACLF of approximately 70% [2], its underlying mechanisms have not been characterized [3–5].

Liver transplantation can effectively treat HBV-ACLF. However, the limited availability of compatible donor livers contributed to the low survival rate. Alternative treatments, including antiviral nucleoside analogues, stem cell transplantation, therapeutic plasma exchange, glucocorticoids, and artificial liver support devices, cannot prevent ACLF but can alleviate the disease, block progression, and improve disease prognosis in the pre- or early LF stage [6–11].

Currently, clinical management of acute exacerbation of CHB is impeded by a lack of reliable early prognostic biomarkers for HBV-ACLF [12]. Serum prealbumin level has been explored as a potential early predictive biomarker for various liver diseases. Mild hepatocyte necrosis is correlated with a high prealbumin level and rapid recovery, while severe hepatitis is associated with a low serum prealbumin level. Notably, a sustained serum prealbumin level of <100 mg/L may be an early indicator of ACLF [13]. The serum thymosin P4 level has also been assessed for its suitability as a prognostic marker for HBV-ACLF [13]. However, both prealbumin and thymosin fail to meet the clinical requirements for biomarkers in terms of sensitivity and specificity because they do not reflect kinetic changes in liver injury.

Given the lack of reliable biomarkers for HBV-ACLF [14], the discovery of early predictive biomarkers is all the more important. The host's immune system exacerbates liver injury; the stronger the immune response, the more severe the liver injury [15–17]. We hypothesized that an early biomarker for HBV-ACLF could potentially be identified among the immunological components.

## 2. Methods

### 2.1. Patients

CHB patients diagnosed with HBV-ACLF from September 2013 to March 2017 admitted to the Department of Infectious Diseases, The Third Affiliated Hospital, Sun Yat-sen University, were recruited for the present study. HBV-ACLF diagnosis was in accordance with the following 18th Asia-Pacific Association of Liver Research consensus criteria [5]: had a history of CHB, acute exacerbation of liver injury, and LF within 4 weeks; serum total bilirubin level (TBIL) ≥ 85 *μ*mol/L; and prothrombin time (PT) ≥ 1.5. All HBV-ACLF patients were administered general supportive treatment, which included hepatocyte protection, antiviral therapy, and plasmapheresis. Precautions were taken to prevent complications. Patients were grouped, based on whether their condition progressed to LF or not during their hospitalization, into LF and non-LF (NLF) groups. The Clinical Trial Ethics Committee of The Third Affiliated Hospital of Sun Yat-sen University approved the study protocol (NCT01627236).

### 2.2. Patient Selection

The inclusion criteria were patients aged between 18 and 60 years; who test positive for HBV DNA and the hepatitis B surface antigen (HBsAg) for >6 months; had obvious or persistent hepatitis symptoms; TBIL >5 times the upper limit of normal (ULN) or TBIL <5 times the ULN and PT activity <60%; and an alanine transaminase (ALT) level of ≥20 times the ULN.

The exclusion criteria were patients who had a history of psychiatric disease, some kinds of ulcer, severe hypertension or diabetes, active tuberculosis, or adrenal hyperfunction; had onset of acute symptoms for over 4 weeks; had complications such as other infections, mild ascites, or gastrointestinal bleeding; receiving therapy with interferon, immunomodulatory drugs, or chemotherapy within the past 6 months; had non-HBV liver disease; had severe systemic disease or tumor other than HBV-ACLF; and were pregnant, lactating, or using estrogen contraceptives.

### 2.3. Sample Preparation and Clinical Indicators

Fresh heparinized blood samples (10 mL) were collected from patients in EDTA tubes to prevent coagulation at admission, on day 3, and in weeks 1 and 2 after admission. The blood samples were centrifuged, and plasma was obtained and stored at −80°C. ALT, TBIL, creatinine levels, and the PT international normalized ratio (PT-INR = PT/prothrombin reference time) were determined at admission, on day 3, and in weeks 1 and 2 after admission. The Model for End-Stage Liver Disease (MELD) score was calculated using the following formula: {3.8 × log (serum bilirubin (*μ*mol/L) × 0.058)} + {1.2 × log (PT-INR)} + {9.6 × log (serum creatinine (*μ*mol/L) × 0.011)} + 6.4 [17].

### 2.4. Detection of Cytokine Levels

We used a commercial CBA kit (BD Pharmingen, USA) to measure plasma cytokines/chemokines (interleukin (IL)-6, IL-8, and IL-10) levels with a FACSCalibur LSR II flow cytometer (BD Biosciences, USA). In brief, we used nine bead populations, each coated with a different cytokine-specific phycoerythrin-conjugated antibody, and measurements were taken at the nine respective fluorescence intensities. Data analyses were performed using the CBA software (BD Biosciences). The inter- and intra-assay coefficients of variation were <10%. The CBA has a lower detection limit of >0.01 pg/mL.

### 2.5. Flow Cytometry

Flow cytometry was used to detect markers, T helper 17 (Th17) and regulatory T cells (Treg), in heparinized peripheral blood. Peripheral blood (200 mL) was incubated for 5 h at 37°C in RPMI 1640 medium with 10% FCS (800 mL) containing phorbol 12-myristate 13-acetate (20 ng/mL) and ionomycin (1 *μ*g/mL). Monensin (1.7 *μ*g/mL) was only added at the 5 h timepoint. Then, the blood cells were fixed, permeabilized, and labeled with phycoerythrin-conjugated IL-17A antibody (eBioscience, USA) or peridinin-chlorophyll proteins-Cy5.5-conjugated cluster of differentiation 3 (CD3) antibody (eBioscience) for flow cytometry. Anti-human forkhead box protein 3 antibody (BD Pharmingen) was used for detection, and CellQuest software (BD Biosciences) was used to analyze the acquired data. Each marker acquisition consisted of at least 10,000 CD4+ cells gated for Tregs and 10,000 CD3+ CD8− cells gated for IL-17.

### 2.6. Statistical Analyses

Data analysis was performed using SPSS v18.0 (SPSS Inc., USA) and GraphPad Prism v5.01. Data with a normal distribution in the NLF and LF groups were expressed as the mean ± standard deviation (SD) and were analyzed using the *t*-test. Data with a nonnormal distribution were expressed as the median and were analyzed using the Mann–Whitney U test. We used Pearson's chi-square test and Fisher's exact test to calculate the differences in percentages and repeated-measures analysis of variance to compare the means among groups.

## 3. Results

### 3.1. Comparison of Baseline Levels of Markers between the NLF and LF Groups

The baseline levels of markers detected in the NLF and LF groups of 100 and 25 patients, respectively, are summarized in Table 1. The period of time between the development of LF and hospital admission for the 25 patients in the LF group is shown in Table 2.

### 3.2. Comparison of Kinetic Changes in ALT and TBIL Levels, PT-INR, and MELD Score between the NLF and LF Groups

#### 3.2.1. Kinetic Changes in ALT

**Figure 1:**
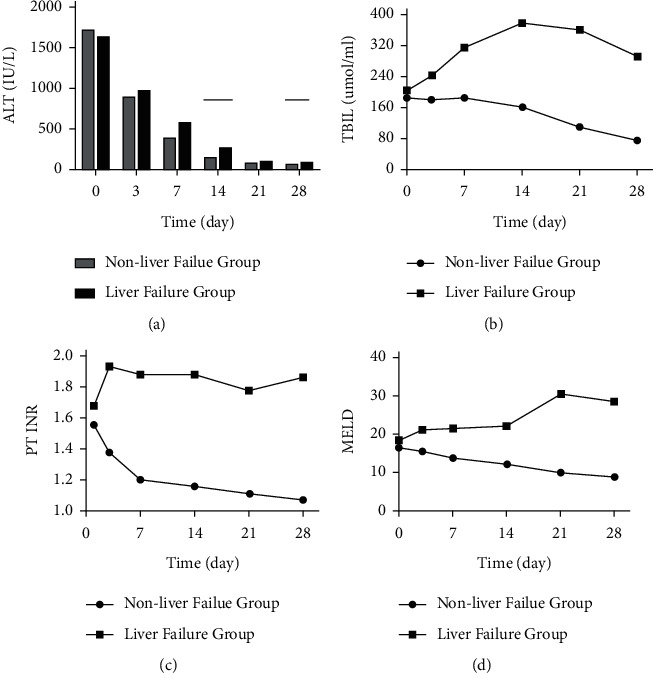
ALT, TBIL, and PT-INR values and MELD score in the NLF and LF groups.

After treatment, we observed a rapid reduction in plasma ALT levels in both the NLF and LF groups. Both groups had comparable ALT levels except in the second (*P*=0.005) and fourth (*P*=0.048) weeks of treatment during which time the NLF group had lower ALT levels (Figure 1(a)).

#### 3.2.2. Kinetic Changes in TBIL Level

The serum TBIL level was slightly elevated in the NLF group and peaked during the first week, before gradually decreasing upon commencement of therapy. For the LF group, serum TBIL level increased gradually and peaked in week 2 before gradually decreasing after treatment commenced. The LF group had a higher mean TBIL level in each week (Figure 1(b); *P* < 0.001 for weeks 2, 3, 4, 6, and 8).

#### 3.2.3. Kinetic Changes in PT-INR

There was a sharp drop in PT-INR in the NLF group and a gradual recovery to normal level by week 4 after treatment. We noted a marked increase in PT-INR in the LF group on day 3 after hospital admission, whereas the NLF group had a significantly lower PT-INR on day 3 after hospital admission (*P* < 0.001). The PT-INR between groups were significantly different (Figure 1(c); *P*=0.008).

#### 3.2.4. Kinetic Changes in MELD Scores

The MELD score in the NLF group gradually decreased after admission but increased in the LF group and peaked in week 3 (Figure 1(d); *P*=0.008). The NLF group had a markedly lower average MELD score from day 3 after admission (*P* < 0.001).

## 4. Comparison of Kinetic Changes in Cytokine Levels between the NLF and LF Groups

### 4.1. Baseline Cytokine Levels

**Figure 2:**
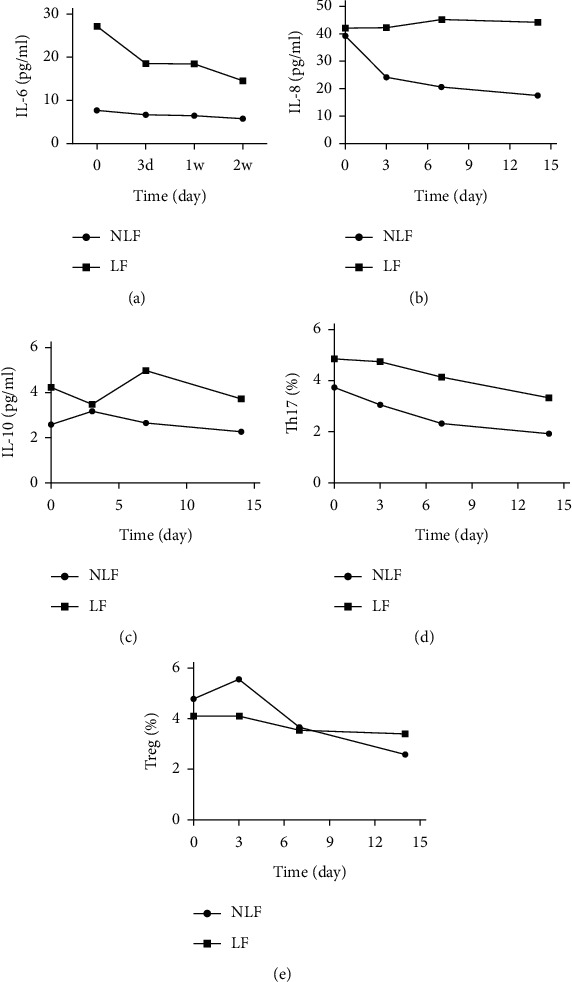
IL-6, IL-8, and IL-10 levels and Th17 and treg numbers in the NLF and LF groups.

The NLF group had significantly lower median baseline IL-6 (NLF vs. LF: 7.84 vs. 27.26 pg/mL; *P* < 0.001) and IL-10 (2.61 vs. 4.23 pg/mL; *P* < 0.04) levels (Table 3). The NLF group had a lower mean plasma IL-6 level than the LF group at admission, which further decreased gradually upon treatment (Figure 2(a); *P*=0.021). The NLF and LF groups had similar plasma IL-8 levels at admission (39.48 and 42.02 pg/mL, respectively). However, IL-8 level in the NLF group significantly decreased from day 3 onward, while it remained high in the LF group during the same period (Figure 2(b); NLF: *P* < 0.01, LF: *P*=0.036). The LF group had significantly higher plasma IL-10 levels than the NLF group at admission (*P*=0.003), but levels were comparable after admission (*P*=0.121) (Figure 2(c)).

### 4.2. Kinetic Changes in the Number of Th17 Cells

The NLF group had a much lower number of Th17 cells than the LF group at admission (*P*=0.001), and the cell number in both groups gradually declined after admission (*P* < 0.001 on days 3 and 7, respectively; and *P*=0.006 on day 14). There were no significant overall changes in Th17 cell numbers between groups (Figure 2(d)).

### 4.3. Kinetic Changes in Number of Tregs

The number of Tregs at admission was similar in the NLF and LF groups but were significantly different after treatment (Figure 2(e); *P*=0.037). After treatment, the number of Tregs in the NLF group sharply increased on day 3 after admission and then gradually decreased. By contrast, the number of Tregs in the LF group remained constant. The NLF group had a significantly higher number of Tregs than the LF group on day 3 (*P*=0.002) and week 2 (*P* < 0.001).

### 4.4. Evaluation of the Predictive Value of the Identified Indicators for Emergent HBV-ALCF

**Figure 3:**
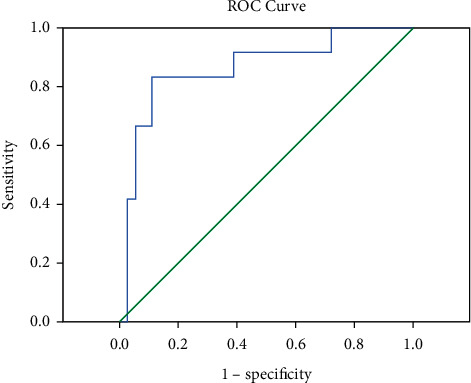
ROC curve to assess the predictive value of baseline IL-6 level for HBV-ACLF development.

We analyzed the associations between age; TBIL; PT-INR; MELD score; IL-6, IL-8, and IL-10 levels; Th17 and Treg cell numbers; and HBV-ACLF development in CHB patients using a Cox proportional risk regression model (forward method). Only baseline IL-6 level was associated with HBV-ACLF development in CHB patients (Table 4; relative risk = 1.082, 95% confidence interval 1.006–1.164, *P*=0.034). The area under the receiver operating characteristic curve (AUC), sensitivity, and specificity for IL-6 level were 82.63%, 83.3%, and 82.9%, respectively (Table 5 and Figure 3; *P*=0.001), suggesting its potential as a prognostic indicator of HBV-ACLF.

## 5. Discussion

Acute exacerbation of liver injury in CHB patients may promote degeneration of a chronically injured liver, thereby inducing fatal LF. It is estimated that about 40–50% of hepatitis B e antigen (HBeAg) positive CHB patients experience acute exacerbation of liver injury upon transition from the HBeAg negative to anti-HBe positive phase [18]. Although the 3-month mortality rate of HBV-ACLF cases can be as high as >50% without liver transplantation [7], there have been few reports of progression from severe exacerbation of CHB to HBV-ACLF. In our study cohort, 33% of patients developed HBV-ACLF, and the 3-month mortality among these patients was 56%, supporting previous reports on the poor prognosis. Several studies have suggested that injury due to an overactive immune system can trigger HBV-ACLF. In CHB patients, the extent of acute exacerbation of liver injury is associated with cytokine levels and immune cell count [15, 16]. Thus, immune response-related markers could be potential candidates for HBV-ACLF prognosis.

In this study, the significantly lower ALT, TBIL, and PT-INR values as well as the MELD score after admission in the NLF group suggested less extensive hepatic necroinflammation in the NLF group. These biochemical and hematological parameters may have clinical value in distinguishing between NLF and LF.

Consistent with previous studies [19, 20], the lower IL-6 and IL-8 levels in the NLF group in this study supported the association with the severity of hepatic inflammation. Moreover, both IL-6 and IL-8 may be implicated in HBV-induced hepatic necroinflammation [19, 20]. Previous studies have suggested the association between IL-10 level and progression of CHB [21]. In this study, we noted a significantly higher anti-inflammatory IL-10 level at admission in the NLF group than in the LF group, indicating a relatively milder immune response and milder hepatic necroinflammation in the former. Th17 and Treg cells are subsets of CD4^+^ T immune cells. Th17 mainly promotes emergent liver inflammation, whereas Tregs negatively regulate the immune response and suppress liver inflammation [22]. We speculate that the lower Th17 and higher Treg cell numbers observed in the NLF group might have been due to the differentiation of Th cells into Tregs. Thus, the lower Th17/Treg ratio in the NLF group might have tempered the immune response and resulted in a milder disease and better prognosis. The difference between our results and a previous report [23] could be due to the enrolled patients being at different stages of LF. The significant differences in immune cells and cytokine levels between the NLF and LF groups suggest that the immune response is a critical contributor of the outcome of acute exacerbation in CHB patients.

Because more than half of the ACLF patients progressed to LF within 3 days after admission, we used different clinical outcomes (NLF or LF) as strain variables and age; baseline TBIL; PT; MELD score; IL-6, IL-8, and IL-10 levels; and Th17 and Treg numbers as covariates in data analysis by the Cox risk regression model. The baseline IL-6 level was found to be a good prognostic indicator of HBV-ACLF, with a high AUC value, sensitivity, and specificity. Most previous studies have not proven the predictive value of IL-6 for HBV-ACLF. Consistent with previous reports [24, 25], our findings suggest the potential for the baseline IL-6 level as a prognostic biomarker for HBV-ACLF for the first time. For example, patients with acute LF or ACLF showed significantly higher hepatic and blood IL-6 levels and more severe hepatic inflammation [24]. IL-6 has also been implicated in HBV-induced hepatic necroinflammation [19]. Furthermore, patients with CHB, cirrhosis, and chronic LF had significantly higher plasma IL-6 level, and more severe liver inflammation [25]. Consistent with these reports, we observed high IL-6 level in the LF group in our study. We also noted that IL-6 level was positively correlated with MELD score, which indicated the severity of inflammatory response and risk of hepatic necrosis. These results support the correlation between IL-6 level and poor patient survival outcome.

Our findings demonstrated the correlation between an elevated IL-6 level and a higher risk for HBV-ACLF. This suggests that the baseline IL-6 level in blood could be a potential early predictor for HBV-ACLF following severe acute exacerbation in CHB patients. Therefore, routine measurement of the plasma IL-6 level at admission is recommended to mitigate progression to HBV-ACLF. Given the small sample size of CHB patients used in our study, the predictive value of baseline IL-6 level requires further clinical verification.

## Figures and Tables

**Table 1:** Baseline characteristics for the NLF and LF groups.

Characteristic	NLF group (*n* = 100)	LF group (*n* = 25)	Statistics	*P*

Age (years)	33.90 ± 8.96	39.83 ± 8.81	*t* = −2.922	0.004
Gender (male/female)	93/7	23/2	*χ* ^2^ = 0.01	0.98
White blood cell count (×10^9^/L)	6.04 ± 1.58	6.32 ± 1.72	*t* = −0.164	0.882
Platelet count (×10^9^/L)	184.14 ± 66.67	172.65 ± 58.37	*t* = −0.397	0.472
ALT (U/L)	1707.26 ± 563.94	1622.13 ± 526.91	*t* = 0.672	0.503
AST(U/L)	983.26 ± 542.33	1046.79 ± 454.98	*t* = −0.530	0.597
TBIL (*μ*mol/L)	187.71 ± 103.96	205.45 ± 100.82	*t* = −0.754	0.452
PT-INR	1.56 ± 0.52	1.68 ± 0.27	*t* = −1.115	0.267
Creatinine (*μ*mol/L)	68.59 ± 12.69	72.84 ± 12.83	*t* = −1.026	0.362
HBV-DNA (copies/mL)	1.02e8±1.92e8	7.92e7±7.17e7	*t* = 0.552	0.582
MELD score	16.66 ± 4.17	18.41 ± 3.64	*t* = −1.892	0.061
Spleen long diameter (mm)	102.76 ± 13.73	108.93 ± 12.64	*t* = −1.243	0.237

Data are expressed as the mean ± standard deviation.

**Table 2:** The period of time between the development of LF and hospital admission for the 25 study subjects in the LF group.

Patient number	1	2	3	4	5	6	7	8	9	10	11	12	13	14	15	16	17	18	19	20	21	22	23	24	25

Days	3	3	5	3	6	4	3	4	3	3	4	5	3	8	3	3	4	3	3	5	3	6	3	4	3

**Table 3:** Baseline cytokine levels in NLF and LF groups.

Parameter	NLF group (*n* = 100)	LF group (*n* = 25)	Statistics *Z*	*P*

IL-6 (pg/ml) (median, IQR)	7.84 (7.02–9.47)	27.26 (19.345–28.575)	−3.74	≤0.001

IL-8 (pg/ml) (median, IQR)	39.48 (23.26–46.50)	42.02 (35.73–71.51)	−1.31	0.19

IL-10 (pg/ml) (median, IQR)	2.61 (1.73–3.88)	4.23 (3.21–6.22)	−2.882	0.04

IQR, interquartile range.

**Table 4:** Cox proportional hazard regression model analysis.

Variable	Wald *χ*^2^	*P*

Age	1.157	0.282
TBIL	0.066	0.798
PT-INR	0.297	0.586
MELD	0.061	0.805
IL-6	4.513	0.034
IL-8	1.490	0.222
IL-10	3.479	0.064
Th17	2.911	0.088
Treg	0.907	0.341

**Table 5:** ROC curve of baseline IL-6 level as a predictor of HBV-ACLF.

Variable	AUC	Cutoff	Sensitivity	Specificity	*P*

IL-6	0.823	15.70	0.833	0.829	0.001

Cutoff value represents the boundary value.

## Data Availability

The data used to support the findings of this study are available from the corresponding author upon request.
